# pXST, a novel vector for TA cloning and blunt-end cloning

**DOI:** 10.1186/s12896-018-0456-8

**Published:** 2018-07-13

**Authors:** Qin Liu, Hui-Jie Dang, Yuan-Hang Wu, Min Li, Yin-Hua Chen, Xiao-Lei Niu, Kai-Mian Li, Li-Juan Luo

**Affiliations:** 1 Hainan Key Laboratory for Sustainable Utilization of Tropical Bioresources, Institute of Tropical Agriculture and Forestry, Hainan University, Haikou, 570228 China; 2 Institute of Tropical Bioscience and Biotechnology, Chinese Academy of Tropical Agricultural Sciences, Haikou, 570228 People’s Republic of China

**Keywords:** PCR product, Cloning, T-vector, Blunt-end ligation, High efficiency

## Abstract

**Background:**

With the rapid development of sequencing technologies, increasing amount of genomic information has been accumulated. To clone genes for further functional studies in large scale, a cheap, fast and efficient cloning vector is desired.

**Results:**

A bifunctional vector pXST has been constructed. The pXST vector harbors a *XcmI-ccdB-XcmI* cassette and restriction site *SmaI*. Digestion the vector with *Xcm*I generates a single thymidine (T) overhang at 3′ end which facilitates TA cloning, and *Sma*I gives blunt end that enables the blunt-end ligation. Multiple products with various sizes were amplified from cassava genome by PCR and each PCR fragment was separately cloned into a pXST using TA cloning and blunt-end ligation methods. In general, the TA cloning gave higher transformation efficiency than blunt-end ligation for inserts with all different sizes, and the transformation efficiency significantly decreased with increasing size of inserts. The highest transformation efficiency (8.6 × 10^6^ transformants/μg) was achieved when cloning 517 bp DNA fragment using TA cloning. No significant difference observed in the positive cloning efficiency between two ligation methods and the positive cloning efficiency could reach as high as 100% especially for small inserts (e.g. 517 and 957 base pairs).

**Conclusions:**

We describe a simple and general method to construct a novel pXST vector. We confirm the feasibility of using pXST vector to clone PCR products amplified from cassava genome with both TA cloning and blunt-end ligation methods. The pXST plasmid has several advantages over many currently available vectors in that (1) it possesses *XcmI-ccdB-XcmI* cassette and restriction site *SmaI*, enabling both TA cloning and blunt-end ligation. (2) it allows direct selection of positive recombinant plasmids in *Escherichia coli* through disruption of the *ccdB* gene. (3) it improves positive cloning efficiency by introducing the *ccdB* gene, reducing the possibility of self-ligation from insufficient digested plasmids. (4) it could be used by high performance and cost-effective cloning methods. Therefore, this dual function vector would offer flexible alternatives for gene cloning experiments to researchers.

## Background

Amplification and cloning of genes are fundamental techniques in the field of molecular biology. DNA amplicons by the polymerase chain reaction (PCR), require to be introduced into the plasmids for propagation, preservation, sequencing before transferred into expression vectors for the following functional characterization. The key step for cloning is to fuse the target DNA fragments into the linearized vectors, and many approaches have been developed to achieve this goal, including “TA” cloning, “blunt-end” cloning, TOPO, Gateway, etc. [1–5].

Generally, the currently available cloning methods can be divided into two categories, according to the critical enzymes catalyzed in the system: ligase-free methods and ligase-dependent methods. Most ligase-free methods achieved the DNA recombinant through DNA pairing and homologous recombination, which requires the whole corresponding systems, including the specific primers and enzymes, such as recombinase or topoisomerase I, thus, increasing the cost and complexity of the methods [6, 7]. Due to the high cost and complicated protocol, hardly can commercially available ligase-free methods meet the requirement for large scale cloning and be used in most laboratories. Comparatively, the ligase-dependent methods are more widely and robustly used, which include cohesive-end ligation, blunt-end ligation and TA cloning [8]. The cohesive-end ligation links the target DNA fragments (inserts) to plasmids through complementary between the inserts and plasmid vectors, resulting in high recombinant efficiency. However, chances are high that the inserts contain multiple cloning sites, increasing the difficulties to choose the appropriate restriction enzymes. The blunt-end cloning walks around such problems by avoiding the digestion of the insert fragments. However new problems arise for the blunt-end cloning because the blunt ends of vectors could be self-ligated, decreasing the available vectors for ligating inserts. TA cloning seems to solve the problem of self-ligation by using the benefit of *Taq* polymerase. The *Taq* polymerase could preferentially add a single adenosine (dA) to the 3′-end of a PCR product which is complimentary to the vectors with a single thymine overhang at 3′-end (T-vector) [9]. Therefore, TA cloning is the simplest and most efficient approach for cloning of PCR products, because the method avoid the requirement of digesting the insert fragments and also the self-ligation problem of vectors.

After the transformation of the recombinant plasmids, an efficient selection method is required for the screening of positive colonies that contain the target inserts. Different strategies, including the white-blue screening, fluorescent protein-based method and introduction of antibiotic resistance and lethal genes, have been described [10–13]. The control of cell death B (*ccdB*) gene encodes a potent inhibitor of gyrase that induces DNA breaking and cell death in the absence of the antidote gene *CcdA* [14]. Such unique characteristic facilitates the positive selection of the recombinant plasmids, because only when the target DNA fragments were inserted into vectors that disrupt the *ccdB* genes, the cells could survive [15, 16].

In this study, we have constructed a novel cloning vector pXST by introducing the *XcmI-ccdB-XcmI* cassette and restriction enzyme site *SmaI* into the plasmid pMD-19 T. The pXST could be used by both TA cloning and blunt end ligation, providing more options when selection of cloning methods. In addition, introduction of *ccdB* genes allows the direct selection of positive colonies and also reduces the self-ligation of blunt-end vectors. Furthermore, the system to catalyze the ligation is cheap and fast. Therefore, this plasmid is expected to be widely used as a high performance and cost effective cloning vector.

## Methods

### Materials

Plasmids pMD-19 T (simple) (TaKaRa, Dalian, China) and pDONOR221 (Invitrogen Corp., CA, USA) served as the backbone and *ccdB* donor vector, respectively. *Escherichia coli* DB3.1 (Invitrogen) was employed to hold the destination vector. *E.coli* strain DH5α was used for the plasmid transformation and cloning. The LA *Taq* polymerase with GC buffer (TaKaRa) and Phusion DNA polymerase (Thermo Fisher, Waltham, MA) was employed to DNA fragment amplification. The PCR products purification and plasmid extraction kits were bought from Omega (Omega, Doraville, USA). T4 ligase and restriction enzyme *Xcm*I and *Sma*I were purchased from Thermo Fisher.

### Primer design

All the primers used in this study were designed by the program Oligo7. The detail information of sequences and corresponding product lengths are listed in Table 1. Two *Xcm*I restriction sites were created by adding sequence (5’ CCAATACTTGTATGG 3′) to the 5′ ends of primers to generate the *Xcm*I-*ccdB*-*Xcm*I cassette.

**Table 1:** Primer sequences used in this study

Name	Primer Sequences	Product size (bp)
*ccdB-F*	CCAATACTTGTATGGgcagactggctgtgtataaggg	487
*ccdB-R*	CCAATACTTGTATGGctccggtctggtaagcacaac	
*ccdBseq-F*	TCTTTTGCTGACGAGAACAG	210 + (insert)
*ccdBseq-R*	CTTTCATCCCCGATATGCAC	
M13F	GTAAAACGACGGCCAGT	126 + (insert)
M13R	CAGGAAACAGCTATGACC	
F1	GGCACAGACAAGATTGAACC	517
R1	AACACTGCAATTCCACGATG	
F2	TCCCGGTTGATTCTTTACGTCT	957
R2	CGCAGTTTCCCATTTGTAATCGTC	
F3	TCCCGGTTGATTCTTTACGTCT	1515
R3	CAATCTTGTCTGTGCCCCGAA	
F4	CTTTGCTTCCTATACCACGAGA	2343
R4	ACTAACACTGCAATTCCACGAT	

The underlined sequence is the specific restriction site for *Xcm*I and the small letters is the complementary sequence for the *ccdB* amplification

### Obtaining the *ccdB* gene

The pDONR221 vector was used as a template for the *ccdB* amplification using ccdB-F and ccdB-R primers. The PCR reaction was performed in 50 μL mixture consisted of 1 ng pDONR221 vector, 0.4 μM of each primer, 0.5 μL *TaKaRa LA Taq*, 2.5 mM dNTP mixture, 10 × LA PCR Buffer I (Mg^2+^ Plus) and an appropriate amount of ddH_2_O. The thermic profile was set as follows: initial denaturing step at 95 °C, 30 s annealing at 56°Cand 30 s extension step at 72 °C, followed by a final extension step at 72 °C for 5 min. The PCR product was then purified by The E.Z.N.A.® Gel Extraction Kit (Omega, cat. no. D2501) following the manufacturer’s instructions.

### pXST construction

The purified product was cloned into the pMD19-T (simple) vector to yield the pXST vector and the ligation was performed according to the manufacturer’s instruction. The ligation mixture was used to transform competent *E. coli* DB3.1 cells. After the overnight culture, several clones were selected to detect the orientation of the inserted *ccdB* fragment using the ccdB-F and M13R primers. The positive clone was cultured in liquid LB medium for overnight. Finally, the pXST plasmid was extracted using the Plasmid Mini Kit (Omega Bio-Tek, USA). The plasmid was digested with *Sma*I to generate the blunt-end fragments and *Xcm*I to create T-vector.

The desired fragments were identified by agarose gel electrophoresis and purified by the Gel Extraction Kit (Omega). The pXST lethality was confirmed by transforming the vector into DB3.1 and DH5α competent cells simultaneously and observing the bacteria growth.

### pXST transformation efficiency and positive cloning efficiency

The DNA fragments of different lengths were amplified from the cassava MeSTP7 genomic sequence (Phytozome accession: Manes.03G180400). All fragments were amplified with or without 3′ terminal A by using the *LA* taq and Phusion DNA polymerase respectively. After the PCR products were purified, the ligation reaction was performed in 10 μL volume using 50 ng linearized pXST, 50 ng purified fragment, 5 U T4 DNA ligase, 1 μL 10 × T4 buffer and 6 μL ddH_2_O. The mixture was incubated at 22 °C for 1 h and then added into 100 μL *E. coli* DH5α chemically competent cells, followed by incubation on ice for 30 min and heat shock at 42 °C for 50 s. All suspended cells were plated on a Luria-Bertani (LB)-agar plate with 100 μg/ml ampicillin. After an overnight culture the colonies were counted to calculate the transformation efficiency by using Calculator (http://www.sciencegateway.org/tools/transform.htm) and analyzed by colony PCR to examine the cloning efficiency. The mean values and standard deviation (SD) were calculated from the measurements of three independent experiments.

## Results

### The construction of the pXST cloning vector

**Fig. 1:**
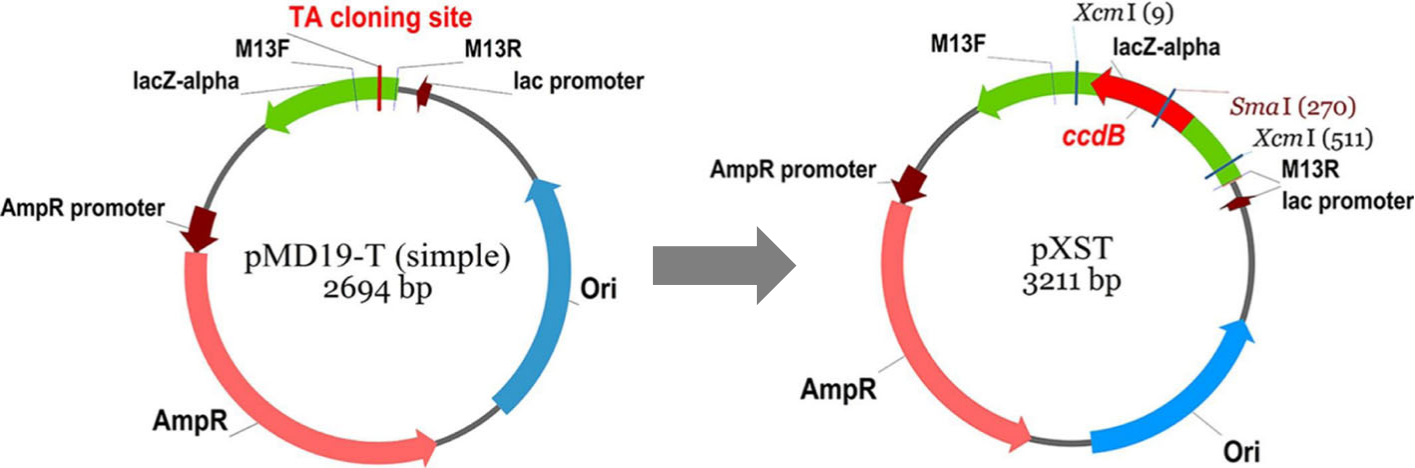
Diagrammatic presentation of the pXST construction. *Xcm*I-*ccdB*-*Xcm*I cassette is derived from the pDNOR221 vector. The recognition sites for *Xcm*I and *Sma*I were indicated as italic. The lethal gene *ccdB* was marked in red color

**Fig. 2:**
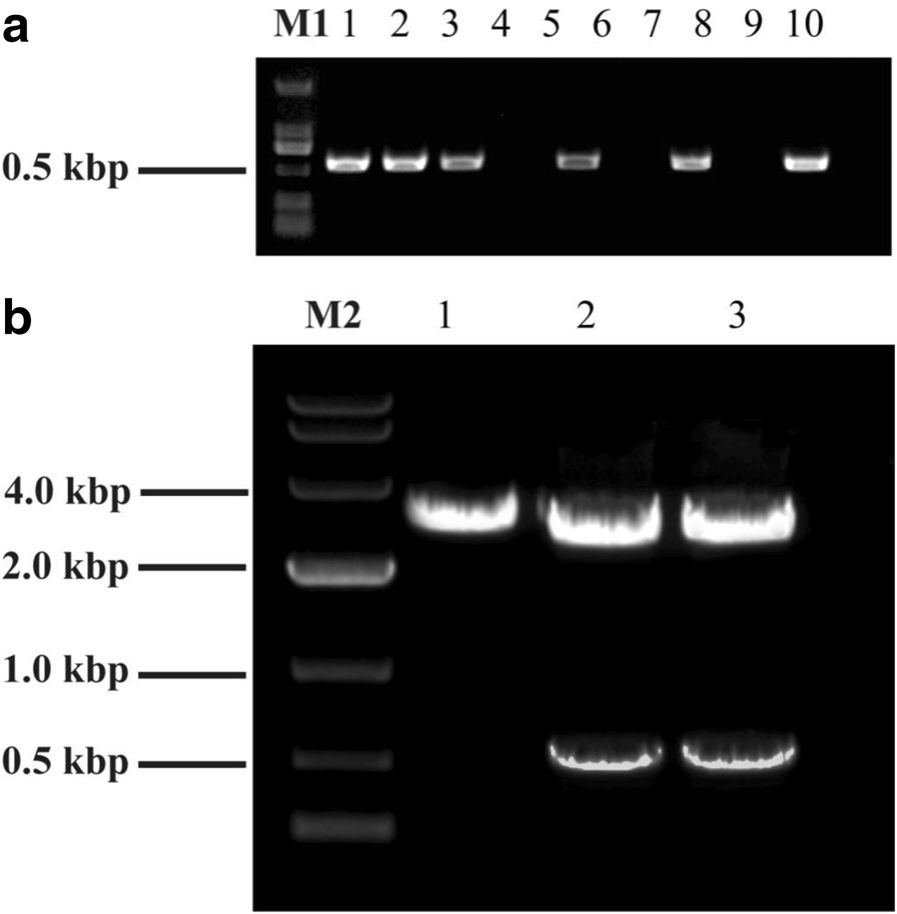
Gel electrophoretogram of colony PCR and restriction. **a** Lane 1–10 shows the results of colony PCR from ten random clones, which were selected for the detecting the insert orientation of *Xcm*I-*ccdB*-*Xcm*I cassette. The light bands indicate positive clones, in which the *ccdB* coding sequence is in the downstream of *LAC* promoter. **b** Plasmid vector pXST was digested with *Sma*I (lane 1) and *Xcm*I (lane 2–3)

**Fig. 3:**
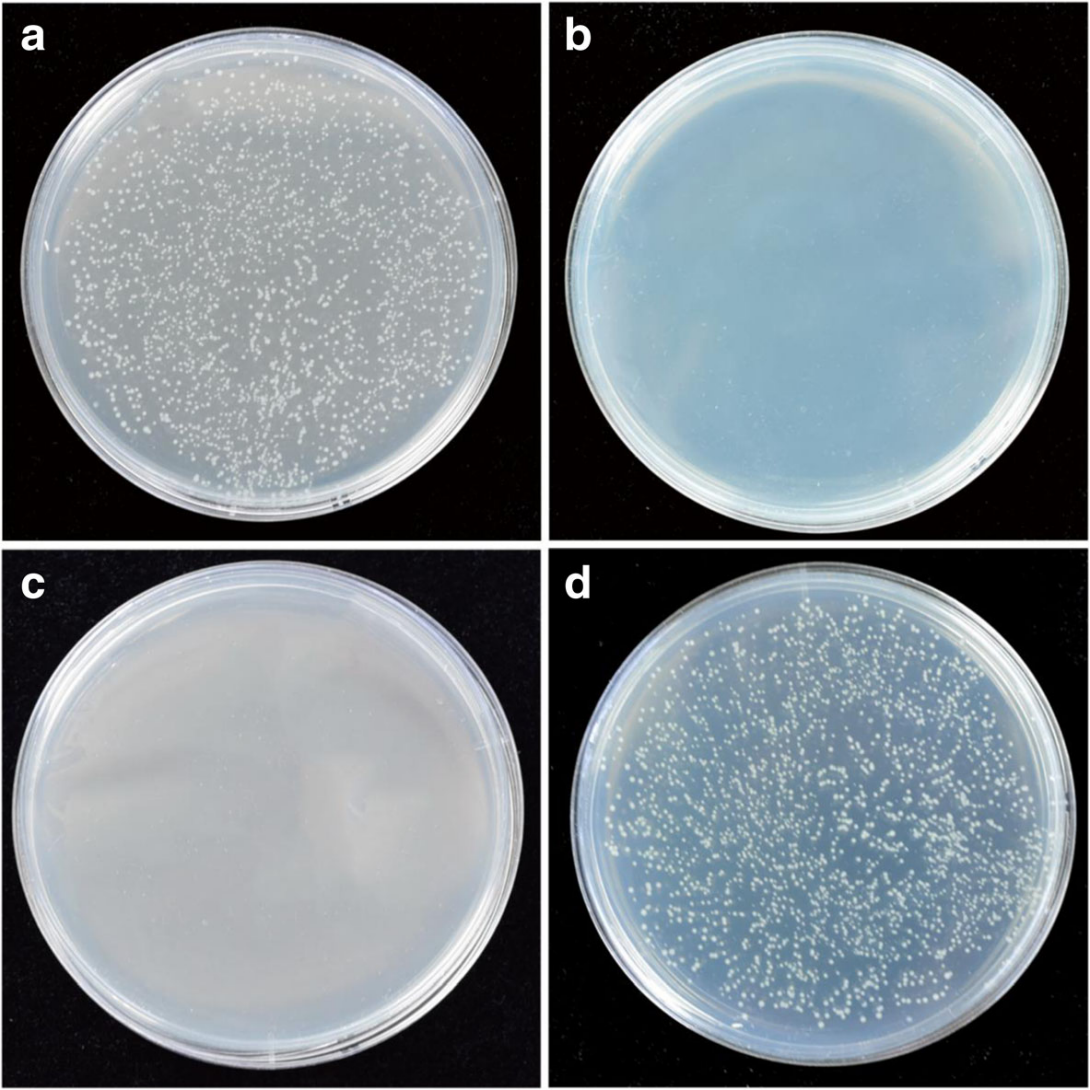
The lethality testing of pXST and pXST-R. **a** Vector pXST was transformed into *E.coli* DB3.1 strain. DH5α strain harboring pXST was grown on the plates with (**b**) or without (**c**) IPTG respectively. **d** Vector pXST-R was transformed into DH5α and the strain survived in LB-plate with IPTG

The pXST vector was constructed followed the flow chart illustrated in Fig. 1. The *XcmI-ccdB-XcmI* cassette was amplified by using the pDONR221 vector as a template. The amplification was catalyzed by the polymerase *TaKaRa LA Taq* which could add an extra adenine to the 3′ end of the PCR fragments. The PCR products were examined by 1% agarose gel. As expected, and the electrophoresis showed a clear strap around the size of 500 bp. The target band was purified and then introduced into the commercial pMD19-T (simple) vector. The orientation of the insert fragment *ccdB* gene was examined by performing colony PCR using the primer pairs, ccdB-F and M13R. After the colony PCR, the products were analyzed by gel electrophoresis (Fig. 2a). There were 6 out of 10 colonies showed a clear band, suggesting that the plasmids showing PCR amplification contain the *ccdB* gene with the expected orientation (pXST) whereas the rest four without amplification have the *ccdB* gene in the reverse orientation (pXST-R). The pXST and pXST-R were verified by sequencing. To examine if insertion of *ccdB* gene in the reverse orientation would also lead to cell lethality, both pXST and pXST-R were transformed into *E. coli* DB3.1 (*ccdB* resistant strain) and DH5α, and the transformants were grew on LB plates with ampicillin selection independently. As showed in Fig. 3, DH5α containing pXST can’t grow on the LB-Amp plates with or without adding IPTG (Fig. 3b, c), suggesting that *ccdB* gene can be well expressed driven by *lac* promoter even at basal level (without IPTG induction), and its expression caused the lethality. Whereas, DH5α with pXST-R can grow on the LB-Amp plate with adding IPTG (Fig. 3d), which indicated that the insertion of *ccdB* in the reverse orientation cannot be expressed, therefore, cannot result in lethality. These results also suggested that the *ccdB* gene fragment in pXST and pXST-R does not contain its own promoter, and its expression requires to be the driven of *lac* promoter. Together, the *ccdB* gene in the pXST is expressed under the control of *lac* promoter and caused cell death in DH5α but not in DB3.1.

### pXST transformation efficiency regarding PCR products

**Fig. 4:**
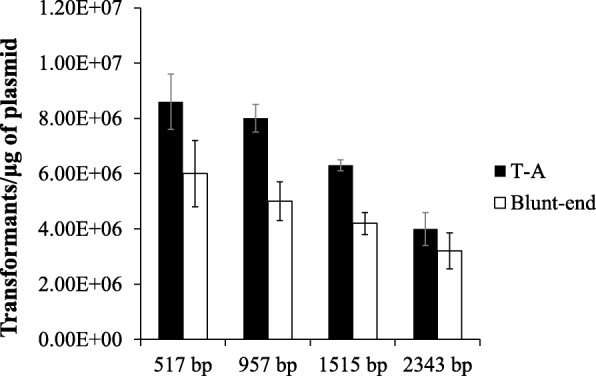
Bacteria transformation efficiency for different sized inserts. Histograms represent average values and error bars mean standard deviations for three individual experiments

**Fig. 5:**
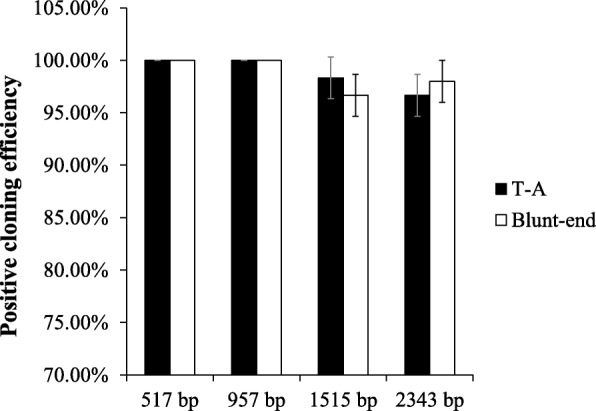
The positive colony efficiency of pXST for different sized inserts. Cloning efficiency is calculated as the ratio of the number of PCR positive colonies to twenty random transformants

Next, we continued to examine the feasibility of using pXST as T-vector and blunt-end vector for conducting both TA cloning and blunt-end ligation. The plasmid pXST was digested with *Sma*I to form a blunt-end vector and with *Xcm*I to generate the T-vector. The digested products were examined by electrophoresis, which showed the bands with the expected sizes (Fig. 2a). Target DNA fragments with different sizes (517, 957, 1515 and 2343 bp, respectively) were amplified from the cassava MeSTP7 genomic regions. In the PCR reaction, two different DNA polymerase was used, and one could add an additional adenine overhang at 3′ end of the PCR products, whereas the other could not. The two kinds of PCR products were cloned into T-vector and blunt end vectors generated from the pXST vector respectively. The transformation efficiency (TE) for different sized inserts were calculated for both TA cloning and blunt end ligation methods. The results showed that the TA cloning generally gives higher TE compared to blunt end ligation for the insert fragments with all different sizes, and the efficiency decreased with the insert size increased (Fig. 4). The highest TE (8.6 × 10^6^ transformants/μg) was obtained when the insert was 517 bp in length. In addition, the positive cloning efficiency was calculated. For each condition, twenty random colonies were chosen to examine the positive insertion by PCR. The positive cloning efficiency was calculated by the ratio of recombinant versus the total number of selected colonies. Statistical analysis showed that the positive cloning efficiency for TA cloning and blunt-end cloning efficiency was both 100% for small inserts (517 bp and 957 bp) (Fig. 5). For the larger inserts, such as 1515 bp and 2343 bp PCR products, the positive cloning efficiency was decreased to 95%. Mean values did not show statistically significant differences between the two ligation methods. These findings highlighted the fact that the pXST cloning system exhibited a strict selection of positive colonies.

## Discussion

Though many commercial kits for DNA cloning are available, the high cost makes it difficult to meet the growing demands for gene cloning in large-scale. In the past years, various strategies have been reported to construct the homemade cloning vectors in laboratories. Most vectors are designed as T-vectors or blunt-end vectors because of theirs convenience in practice.

T-vector could be constructed by either adding a single thymidine residue to a blunt-end plasmid by transferase [17] or by restriction endonucleases, such as *Hpb*I [18], *Abd*I [19], *Bfu*I [20] and *Xcm*I [21], which can recognize restriction sites containing variables nucleotide sequences, which provide a feasible way to construct T-vectors. Among above enzymes, *Xcm*I has been widely used, because pUC-based plasmids which are the most popular vector series do not contain the restriction sites recognized by *Xcm*I. Therefore, addition of *Xcm*I site to the pUC-based vectors would not disrupt their original features. However, T-vectors generated from restriction digestion would have self-ligation problems come from insufficiently digestion, especially when the size of the fragment between the two *Xcm*I is too small [21]. Therefore, inserting a relative large DNA fragment within the two *Xcm*I sites seems to be an alternative choice to solve the problems mentioned above when designing the vector [22]. In the present study, we constructed the *Xcm*I-*ccdB*-*Xcm*I cassette with length of 487-bp which is large enough for the *Xcm*I digestion. Furthermore, the *ccdB* lethality can guarantee that the transformants containing self-ligation vector would not survive, adding an additional layer of selection.

The blunt-end vectors have been frequently used for cloning DNA fragments since the DNA polymerases used to amplify DNA fragments have the high-fidelity due to the 3′-5′ exonuclease activity and generate blunt-end fragments after the PCR reactions. Blunt-end vectors can be generated by PCR or enzymes digestion. *EcoR*V and *Sma*I are the most popular endonucleases utilized to digest vector and produce blunt-end. Since the linearized vector and DNA fragment are both blunt-end in the T4 ligation reaction mixture, it causes the problem of vector self-ligation, reducing the possibility of successful recombinant and bringing great inconvenience in the following positive colonies screening. Although the dephosphorylation can be performed to minimize self-ligation of the linearized vector [23], it is still not an ideal solution as it is time consuming and costly. In this study, we introduced the *Sma*I site within the *ccdB* gene. Therefore, if the vectors are self-ligated, the expression of *ccdB* genes will result in cell death, reducing the possibility of the colony containing the self-ligated empty vectors, thus increasing the positive colony efficiency.

The pXST vector transformation efficiency regarding different sized PCR products was calculated by two ligation methods, TA cloning and blunt-end cloning. Generally, transformation efficiency is determined by a variety of factors, including the quality of competent cells, the molar ratio between the insert fragment and plasmid, and the ligation efficiency of the reaction mixture, etc. [24]. As high efficiency of transformation is not critical for cloning and subcloning purposes, a simple strategy that that can meet the minimum requirement for transformation without complex computation for the ratio of vector versus insert is more desirable. In this study, the transformation efficiency ranging from 3.0 × 10^6^ to 8.3 × 10^6^ transformants/μg plasmid, was sufficient to meet the requirements of gene cloning and comparable to pELMO, which is a pUC18-based vector for TA cloning [25]. In addition, such efficiency could be reached even with equivalent amount of insert and pXST (50 ng for each, 1:1 ratio), increasing the convenience for practical experiments. The transformation efficiency of TA cloning is much higher than that of blunt-end ligation, which is due to the fact that T-A complement between the T-vector and the PCR fragment is easier for T4 ligation reaction to catalyze. For an ideal cloning vector, it should have a high positive cloning efficiency in addition to high transformation efficiency. The cloning efficiency of different size fragments between the two ligation methods was also tested in this study. The cloning efficiency was as high as 95% for both methods and there was no significant difference between the two methods. These results also supporting the idea that the introduction of *ccdB* can reduce the possibility of self-ligation of vector.

Researchers usually require different plasmids to meet cloning requirements. In the present study, we constructed a bifunctianal vector pXST by combining *Xcm*I, *Sma*I and *ccdB* genes, which can be digested into linearized T carrier or blunt end vector. The vector pXST utilizes the *ccdB* gene as a positive selection maker, saving the time in screening the positive colonies. For cloning vectors, they should have characteristics that allow the gene to be easily inserted into or removed. The pXST is designed without redundant cloning sites that would allow researchers to add any desired restriction enzyme sites to facilitate the subclone. In this study, the pXST was just designed for gene cloning, but the *Xcm*I-*ccdB*-*Xcm*I cassette within the vector, which is proved to be a useful element in vector modification and deserved to be further used in the design of expression vectors. It would be serviceable in the plasmid constructions for various purposes, including gene over-expression, sub-cellular location, protein expression, etc.

## Conclusions

In conclusion, a novel cloning vector pXST with high efficiency and low background is constructed in this study. The mechanism is easy to understand, and technique is simple to carry out. It is suitable for any lab to build this dual functional vector in with low cost.

## Abbreviations

Amp: Ampicillin
*ccdB*: Control of cell death B
dA: Adenosine
IPTG: β-D-1-Thiogalactopyranoside
LB: Luria–Bertani
PCR: Polymerase chain reaction
TE: Transformation efficiency
